# Genetics of Congenital Heart Disease: A Narrative Review of Challenges and Strategies in Identifying Novel Genes

**DOI:** 10.7759/cureus.91674

**Published:** 2025-09-05

**Authors:** Eteesha Rao, Srinivas Annavarapu

**Affiliations:** 1 Department of Medical Education, Newcastle University, Newcastle, GBR; 2 Department of Paediatric Histopathology, Alder Hey Children’s Hospital, Liverpool, GBR

**Keywords:** congenital heart disease, gene regulation, genetics, genomics, signalling pathways, transcription factors

## Abstract

Congenital heart disease (CHD) is the most common congenital anomaly. While surgical and interventional advancements have improved survival, the management of associated complications and comorbidities remains complex and would benefit from a personalised approach that more accurately predicts individualised risks and prognoses. Recently, next-generation sequencing has uncovered diverse genetic factors, including epigenetic modifications, somatic mosaicism and regulatory non-coding variants. Despite these advances, challenges persist in translating genomic data into clinical risk prediction and therapeutic guidance. Experimental approaches, such as patient-derived induced pluripotent stem cells (iPSCs) and single-cell sequencing, offer promising preclinical models for interpreting novel variants. Integrating genomic insights into clinical practice requires multidisciplinary team collaboration. The clinicians need to keep themselves abreast with current genomic advancements to optimise customised, individualised care to improve future outcomes for patients with CHD. We aim to provide an overview of the advances in genomic technologies linking the genetics of cardiac development with the pathogenesis of CHD for a better understanding of molecular-morphological correlation.

## Introduction and background

Congenital heart disease (CHD) is the most prevalent congenital malformation, occurring in approximately 1% of live births worldwide (8-10 per 1,000) [1,2]. Among pregnancies undergoing a termination of pregnancy for fetal anomalies (TOPFA), the relative contribution of CHD is markedly higher. Recent epidemiological studies report that 10%-20% of structural anomalies leading to a termination of pregnancy (TOP) involve CHD, with severe forms such as hypoplastic left heart syndrome (HLHS), atrioventricular (AV) septal defects and conotruncal anomalies being most frequently implicated [3,4]. Data from China demonstrate a prenatal CHD prevalence of 7.4 per 1,000 pregnancies, with ventricular septal defect (VSD) (29%) and tetralogy of Fallot (TOF) (14%) as the most common diagnoses [5]. Northern England surveillance reports a total prevalence of ~86 per 10,000 births (0.86%), with around 5% of CHD-affected pregnancies ending in TOPFA [6]. Across Europe, critical CHDs (requiring intervention in infancy) constitute 20%-25% of all CHDs (one per 500 births), and overall prevalence is estimated at eight per 1,000 total births [7]. Collectively, these findings emphasise the disproportionately high representation of CHD in fetomaternal medicine settings, reflecting advances in prenatal detection, the severity of many malformations and their substantial impact on parental decision-making and perinatal outcomes.

Several factors contribute to the underestimation of the true burden of disease. Importantly, severe cardiac malformations may result in intrauterine fetal demise, or stillbirth, before a formal diagnosis can be established. In many parts of the world, particularly where comprehensive perinatal surveillance is lacking, such cases remain undocumented. Additionally, mild or asymptomatic forms of CHD, such as bicuspid aortic valve and small atrial or ventricular septal defects, often go undetected during infancy, in the absence of routine echocardiographic screening. These defects may only be identified later in life, incidentally during evaluations for unrelated conditions, leading to underrepresentation in paediatric registries [1]. Inconsistencies in diagnostic criteria, inclusion thresholds and follow-up durations across epidemiological studies further contribute to variations in the reported prevalence rates [8-11]. While some registries may include only haemodynamically significant defects, others may record all structural anomalies, irrespective of their clinical severity. These methodological differences hinder accurate cross-regional comparisons and the accurate estimation of the epidemiology of CHD globally. Recent large-scale genomic studies have further advanced the field; for example, Sierant et al. (2025) identified 60 dominant CHD-associated genes from more than 11,000 probands, underscoring both inherited and de novo contributions to disease pathogenesis [12].

Accurately defining this burden is important, not only for health policies and resource allocation. Recent advances in prenatal diagnostics, surgical interventions and postoperative care have improved survival. A recent systematic review and meta-analysis reported pooled survival rates of 87% at one year and 85% at five years following congenital heart disease surgery, with outcomes significantly higher in high-income countries compared to earlier decades [13]. In spite of this, CHD still remains an important cause of infant morbidity and mortality. Recent advancements in genomic research in CHD have uncovered diverse factors, including novel gene mutations, epigenetic modifications, somatic mosaicism and regulatory non-coding variants that allow for the translation of genomic data for individualised clinical risk prediction, prognostication and developing personalised medical management [2,9].

Regulatory non-coding variants include enhancer, promoter and splice-regulatory changes that disrupt transcription factor binding or chromatin looping, thereby altering the timing and location of cardiac gene expression. For example, enhancer variants in TBX5 and GATA4 have been shown to mis-specify cardiogenesis, contributing to atrial and ventricular septal defects. Identifying CHD-causing genes remains difficult due to extensive genetic heterogeneity, incomplete penetrance and variable expressivity (as seen with NKX2-5 and GATA4 variants), oligogenic inheritance and gene-gene interactions and the technical difficulty of detecting tissue-restricted mosaicism and non-coding regulatory variants with routine sequencing [9].

The primary aim of this review is to provide an overview of the recent advances in our understanding of the genetic basis of CHD. We also wish to explore issues that confound the discovery of novel CHD-causing genes and explore strategies to improve the ‘hit rate’, proportion of cases in which genomic sequencing identifies a likely pathogenic or pathogenic variant for finding potential genes causing CHD, eventually enabling better genetic counselling and improved personalised medicine. Molecular-morphological correlation involves mapping variant classes and genes to embryological stages, supported by single-cell and spatial atlases of human cardiac development, and cross-referencing these to lesion-specific clinical phenotypes.

## Review

Methods

Literature Search Strategy

This review was conducted as a narrative review rather than a systematic review or meta-analysis. The purpose of a narrative review is to synthesise and critically interpret existing literature, highlight advances and provide context for future research directions, rather than exhaustively retrieve or statistically analyse all available studies.

A comprehensive literature search was conducted using the PubMed database to identify peer-reviewed articles on the genetic basis of CHD. The search was performed using a combination of Medical Subject Headings (MeSH) and free-text keywords, including ‘congenital heart defect’ AND ‘genetics’ OR ‘genomics’ OR ‘transcription factors’ OR ‘signaling pathways’ OR ‘gene regulation’.

Two reviewers (ER and SA) independently screened all titles and abstracts; disagreements were resolved through discussion, and if consensus could not be achieved, the article was excluded from the review.

Inclusion Criteria

Articles were selected based on their relevance, originality and contribution to understanding the genetic and developmental basis of CHD and emerging technological applications. Seminal works were included to provide a historical perspective, while recent high-impact studies were prioritised to capture ongoing developments. References were curated to align closely with the statements made in the text, and tangential citations were excluded. As a narrative review, Preferred Reporting Items for Systematic Reviews and Meta-Analyses (PRISMA) flow diagrams, formal bias analyses and statistical syntheses were not applicable.

Only articles published in peer-reviewed journals indexed in PubMed were shortlisted. Studies focusing on the genetic basis of CHD, including investigations of transcription factors (TFs), signalling pathways, gene regulatory networks and non-coding variants, were selected. The articles included original research, systematic reviews, meta-analyses and high-quality narrative reviews. Only studies involving human subjects or in vitro systems (e.g. stem cell-derived cardiomyocytes) relevant to CHD genetics were included. Articles also included studies utilising genomic approaches, such as whole-exome sequencing (WES), whole-genome sequencing (WGS), single-cell RNA sequencing (scRNA-seq) and/or functional genomics tools in CHD.

Exclusion Criteria

Articles not related to genetics or genomic investigations in CHD were excluded. Case reports and small case series (n < 5), unless reporting novel genetic variants of high significance, were excluded. Studies published in languages other than English were not included. Finally, conference abstracts, editorials, expert opinion pieces or articles lacking primary data or systematic methodology were also excluded.

Aetiology of CHD

**Figure 1:**
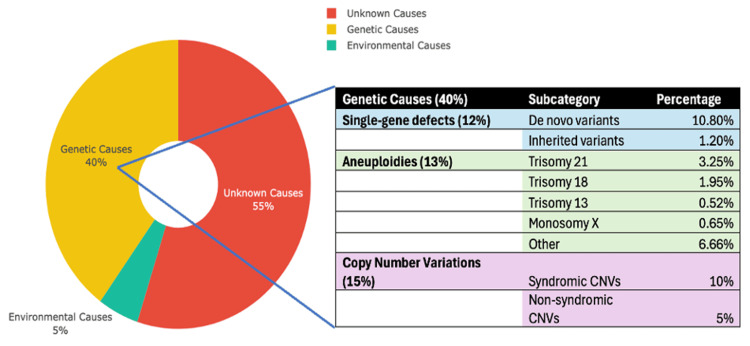
Causes of CHD with a focus on genetic causes The pie chart shows the proportion of each genetic cause, with a table providing a further breakdown of causes within each category [1,2,8] CHD, congenital heart disease; CNVs, copy number variants

The underlying aetiology of CHD is complex and is thought to include both genetic and environmental factors [1,2,9]. Recent advances have identified underlying causes for 45% of CHDs, including genetic (40%) and environmental factors (5%) (Figure 1) [1,2,9,14-16]. In contrast to the general population, there is a high concordance of CHD in monozygotic twins with higher incidence in consanguineous parents, strongly underpinning the underlying genetic basis [7,14-16]. In addition, certain genetic syndromes are recognised to be frequently associated with particular phenotypes of CHD, for example, conotruncal defects in DiGeorge syndrome [17,18], atrioventricular septal defect in trisomy 21 [18,19] and tubular hypoplasia/coarctation of the aorta in Turner syndrome [18,20], all indicating a strong genetic element to CHD.

Around 60% of the CHDs may be related to ciliopathies [18]. All classes of genetic variation, including chromosomal aneuploidies, copy number variants (CNVs) [21] and rare and common de novo and inherited single-nucleotide variants (SNVs), contribute to CHD [9,22-25]. A small number of CHDs are thought to be related to environmental causes, including maternal diabetes [26,27], obesity [28], teratogens [29], drugs [30,31], alcohol [32], maternal fever [33], placental insufficiency [34] and dietary deficiencies of vitamin A [35] and folate [36]. The cause for the remaining 55% CHDs is still elusive and is presumed to be multifactorial in nature [9,36].

CHDs encompass a wide array of structural abnormalities of the heart and great vessels, resulting from disruptions in embryonic cardiac development. Heart morphogenesis is driven by a precise interplay between transcription factors, signalling molecules and environmental cues [2,22-25,37-42]. Genetic mutations or regulatory dysfunctions in these pathways can impair cardiac lineage specification, cell migration and morphogenesis, leading to clinically significant defects.

Stages of cardiac development

Cardiac Specification

Soon after the completion of gastrulation, cardiac development commences, somewhere between days 15 and 21 of human development. In this stage, from the primitive streak, embryonic mesodermal cells migrate to form the cardiac crescent that eventually transforms into the primary heart field (PHF). PHF primarily forms the bulk of the left ventricle and portions of the atria. This specification is regulated by a series of key transcription factors, including NKX2-5, GATA4, and MEF2C. Other signalling pathways, such as Wnt, bone morphogenetic protein (BMP), and fibroblast growth factor (FGF), also play crucial roles in regulating fate decisions. Disruption in these pathways, especially with mutations in NKX2-5 or GATA4, can interfere with this process and cause atrial/ventricular septal defects and conduction system anomalies [2,37-40].

Heart Tube Formation and Looping

In the third to fourth gestational week, PHF-derived cardiac crescent fuses in the midline to form the linear heart tube that further undergoes rightward looping, a critical morphogenetic movement that establishes cardiac polarity. During this time, the second heart field (SHF) contributes additional progenitor cells to form the right ventricle and the outflow tract; key genes controlling this process include *TBX1*, *FOXC1/2* and *ISL1*, which control the proliferation and elongation of the heart tube. On the other hand, the left-right asymmetry is regulated concurrently by another set of genes, *LEFTY*, *PITX2* and *NODAL*. Aberrations in these pathways can lead to CHD; for example, *ZIC3* mutations cause visceral heterotaxy syndromes; TBX1 deletion causes 22q11.2 deletion syndrome/DiGeorge syndrome, associated with conotruncal anomalies such as tetralogy of Fallot (TOF) and interrupted aortic arch [2,18,37-40].

Chamber Formation and Septation

During gestational weeks 4-8, the linear cardiac tube expands into distinct atria and ventricles; endocardial cushions form atrial and ventricular septa and the AV valves. This critical phase depends upon a cascade of transcriptional regulators, including TBX5, CRELD1, NOTCH1, and GATA4, which coordinate the formation of the atrial and ventricular septa, as well as the atrioventricular valves. Mutations in these genes can cause CHDs; for example, TBX5 mutations are observed in Holt-Oram syndrome, resulting in atrial septal defect (ASD)/VSD, along with limb defects; NOTCH1 mutations are associated with AV septal defects and bicuspid aortic valve, and CRELD1 mutations have been linked to AV canal defects [2,18,37-40].

Outflow Tract and Valve Development

Between six and 10 gestational weeks, the truncus arteriosus, the common arterial trunk, needs to be separated into the ascending aorta and the pulmonary artery by the neural crest and SHF-derived cells. The respective valve formation also begins now. Genes such as *JAG1*, *NOTCH1*, *TGF-β* and *BMP* signalling are involved in this phase of cardiac development. *JAG1* mutations result in Alagille syndrome, which shows pulmonary valve and artery defects; also, deletions in 22q11.2 disrupt neural crest migration, eventually resulting in a range of conotruncal defects. Structural proteins such as elastin and transcription factors such as NFATC1 also contribute to the shaping and remodeling of valve structures. Disruptions during this phase often lead to outflow tract defects such as persistent truncus arteriosus, double outlet right ventricle or the transposition of the great arteries [2,18,37-40].

Maturation and Remodeling

Post structural development, the heart goes through a series of morphological and functional maturation, including myocardial compaction, the alignment of the conduction system, and the formation of coronary vessels from the epicardium, requiring genes such as SCN5A and MYH6. Although less understood than earlier stages, this phase is critical for establishing efficient, synchronised cardiac function. Errors in remodeling can lead to persistent myocardial non-compaction, arrhythmias or late-onset valvular disease [21,37,43].

Signalling Pathways and Transcription Factors in Cardiac Development

Many signalling pathways and transcription factors regulate gene expression patterns that are critical for the proper development of cardiac structures. Several families of TFs have been implicated in human CHDs [18,38].

NKX2-5

NKX2-5 is a homeobox transcription factor expressed early in cardiac progenitor cells and is considered to be the master regulator as it regulates genes involved in cardiac looping, septation and conduction [21]. Mutations in NKX2-5 have been associated with ventricular septal defects (VSD), hypoplastic left heart syndrome (HLHS), atrial septal defect and tetralogy of Fallot (TOF) [18,37-40].

GATA Family

These zinc-finger transcription factors are crucial for cardiomyocyte differentiation and myocardial growth. Mutations in GATA4 have been implicated in patients with septal defects, atrial and ventricular. GATA factors are expressed in the developing heart where they cooperate with TBX5 and NKX2-5 in regulating other cardiac-specific genes, including natriuretic peptides (NPPA and NPPB) and cardiac myosin heavy chain (MYH6 and MYH7) [18,37].

T-box Transcription Factors (TBX1 and TBX5)

T-box proteins control outflow tract and chamber formation. TBX1 is strongly linked to 22q11.2 deletion syndrome (DiGeorge syndrome), which includes conotruncal heart defects; TBX1 is also expressed in the pharyngeal endoderm, mesoderm and ectoderm [17,18]. TBX5 is highly expressed in the forelimb buds and the developing heart, and mutations in this gene cause Holt-Oram syndrome [18,37-40]. TBX20 is a transcription factor whose mutation is implicated in atrial septal defect type 4 (ASD4). It also regulates endocardial cushion proliferation and chamber septation. Mutations in TBX20 have been associated with valvular dysplasia, dilated cardiomyopathy and left ventricular non-compaction, underscoring its broader developmental and clinical significance [19].

Cell Signalling Pathways

Notch signalling is essential for endocardial cushion formation and valve morphogenesis. NOTCH1 signalling in endocardial cells triggers endothelial-to-mesenchymal transition, enabling endocardial cushions to populate with mesenchymal cells. This process is essential for valve elongation and stratification, and Notch dysregulation leads to bicuspid aortic valve and hypoplastic left heart syndrome (HLHS). The dysregulation of this pathway has been linked to left-sided heart lesions such as bicuspid aortic valve and HLHS [37-40].

Wnt/β-catenin pathway has biphasic effects on heart development, promoting cardiac progenitor expansion in early stages and suppressing differentiation later. Canonical Wnt/β-catenin signalling is active in the mesoderm during gastrulation and cardiac mesoderm specification, where it promotes progenitor proliferation. In later stages, particularly during chamber differentiation and outflow tract septation, Wnt signalling must be suppressed to permit cardiomyocyte maturation. Dysregulation at either phase contributes to myocardial hypoplasia or outflow tract malformations [37].

Bone morphogenetic protein (BMP) signalling, particularly BMP2 and BMP4, drive myocardial specification and septation. BMP2/4 variants associated with CHD have been reported primarily in animal models, but human studies have linked BMP4 mutations and CNVs to atrioventricular septal defects (AVSDs) and left-sided malformations [37]. Fibroblast growth factor (FGF) signalling controls the proliferation and migration of second heart field cells. FGF8 secreted from the pharyngeal endoderm regulates the proliferation and migration of second heart field progenitors into the outflow tract. The disruption of FGF8 or FGFR1/2 impairs this migration, leading to outflow tract shortening, misalignment and conotruncal defects such as truncus arteriosus and double-outlet right ventricle [2,37]. Finally, the sonic hedgehog (Shh) pathway is vital for second heart field development, influencing outflow tract rotation and septation. Mutations affecting the Shh pathway are associated with pulmonary atresia and double-outlet right ventricle [2,37].

Transcription factors interact with various signalling pathways to form intricate gene regulatory networks. For example, GATA4 and NKX2-5 cooperatively activate cardiac structural genes, while BMP signalling enhances the activity of MEF2 proteins through phosphorylation. GATA4-NKX2-5 physical and functional interactions have been confirmed in both mouse models and human cardiomyocytes, with human genetic studies showing digenic inheritance patterns involving GATA4 and NKX2-5 variants in families with atrial and ventricular septal defects. Such dynamic interactions ensure that cardiomyocytes differentiate and organise in a controlled manner. The precise timing and location of transcription factor and signalling pathway activity (e.g. NKX2-5 in early cardiac progenitors, Notch in valve endocardium and Shh in the second heart field) ensure the ordered progression of morphogenetic events. Disruption at inappropriate times or locations alters lineage specification and structural outcomes [18,37-40].

The systematic understanding of embryonic heart development and a deep knowledge of the spatiotemporal interrelationships of these genetic regulatory networks are crucial to understanding the genetic basis of CHD [18]. Developmental biology provides the most robust framework for grouping CHD phenotypes. Rather than relying solely on descriptive or anatomical categories, classification can be systematically anchored to the embryological stage at which cardiac morphogenesis is disrupted. For example, defects arising from disturbances in early left-right patterning (e.g. heterotaxy) can be distinguished from those linked to endocardial cushion formation (atrioventricular septal defects), outflow tract septation (tetralogy of Fallot and truncus arteriosus) or valve stratification (bicuspid aortic valve). Aligning phenotypes to embryonic stages ensures biological homogeneity within study groups and increases the likelihood of detecting shared genetic determinants. Advances in single-cell genomics and functional studies continue to refine our understanding of these pathways, offering the potential for early diagnosis, targeted therapies and genetic counselling [41].

Challenges in discovering new CHD-causing genes

Phenotype Heterogeneity

Unlike other genetic diseases, such as inborn errors of metabolism that have a defined phenotype associated with a single gene mutation, CHD is a complex genetic disorder that results from abnormalities in normal cardiac development during the period of organogenesis [9,19-24]. Therefore, very few CHDs follow the classic Mendelian inheritance pattern. What makes things even more complex is that a defect in one gene may cause multiple congenital defects, or the dysregulation of multiple genes may disrupt a developmental field because of gene redundancy [19-24]. Furthermore, even if a putative gene mutation is thought to be responsible for a CHD in an affected family, there may be a wide variation in the phenotypes of CHDs in the family members, as there may be partial penetrance with variable phenotypes [2,9].

Genetic Variation

Another factor that complicates the issue is natural human genetic variation, which makes the interpretation of genetic data challenging. Single-nucleotide polymorphism (SNP) may result in a difference in DNA length relative to the control. Similarly, CNVs may occur due to large insertions or deletions of DNA, causing genetic variation [2,3,5,9,38,39,44,45]. While genetic testing has become an invaluable tool in the evaluation of congenital conditions, it is not infallible; false-positive and false-negative results may occur, and in some cases, testing does not provide a definitive diagnosis [46].

Variants of unknown significance (VUS) are commonly identified, which makes interpretation complex, requiring in-depth inputs of bioinformatics experts, geneticists and clinicians with expertise in CHD [8,19-23,38]. The genes involved in heart development are conserved across species during evolution, allowing us to utilise animal models when investigating CHDs and their causes. Mouse models are widely used to study cardiovascular development because they share a high degree of sequence conservation with humans and recapitulate human cardiac development. While mouse models are invaluable for studying cardiovascular development, caution must be applied when using their phenotype data for variant classification, as genotype-phenotype correlations may differ between species and thus may not provide definitive evidence for human pathogenicity [18].

Functional validation is most informative when carried out in a staged, hierarchical pipeline. Candidate variants should first be prioritised through in silico predictions and segregation analyses, after which in vitro assays, such as reporter gene activity or clustered regularly interspaced short palindromic repeats (CRISPR)-engineered induced pluripotent stem cell (iPSC)-derived cardiomyocytes, can be used to examine direct effects on gene regulation, protein function or electrophysiological properties. Variants that demonstrate plausible functional disruption in vitro can then be modeled in vivo using zebrafish or murine systems to assess their developmental and structural consequences. This progressive workflow minimises false leads, balances efficiency with rigour and allows the integration of molecular-level findings with organismal phenotypes.

Recent research utilising single-cell RNA sequencing (scRNA-seq) and various functional genetic approaches is beginning to reveal the intricate network of transcription factors and signalling pathways involved in heart development. By capturing transcriptomic signatures of endocardial, myocardial, and neural crest-derived cell populations at these stages, scRNA-seq provides high-resolution insights into disease-relevant gene expression [37-40]. Meanwhile, whole-genome sequencing has highlighted the role of non-coding de novo mutations within the complex genetic framework of CHDs. However, the precise impact of these mutations on gene transcription and their contribution to disruptions in cardiac development remains largely undefined [41].

Non-coding Variants

A recent study by Xiao et al. (2024) suggested that non-coding regions of the genome may play a significant role in the development of CHD [47]. Xiao et al. (2024) analysed data from over 13,000 individuals with CHD and more than 18,000 of their family members. Among these, over 3,000 patients underwent whole-genome sequencing (WGS), including 750 trios comprising affected individuals and their parents. After applying robust statistical methods to filter out background variation, the team identified ~7,000 non-coding DNA variants in CHD patients [47]. From this pool, 403 variants were found to disrupt the function of transcriptional enhancers, making them strong candidates for contributing to CHD. To test their functional significance, the researchers introduced 10 of these non-coding variants into human stem cells at precise genomic locations. When these cells were differentiated into cardiomyocytes, four of the 10 variants led to changes in the expression of nearby genes, some of which are known to be associated with CHD, and in certain cases, the mutations impaired enhancer function. These findings provide preliminary but compelling evidence that non-coding genetic variants can influence cardiac development and may contribute to the underlying CHD [47].

Although WES and WGS allow for the discovery of a large number of CNVs and single-nucleotide variations (SNVs) in regulatory and non-coding regions of the entire human genome, establishing a link between non-coding genetic variation and CHD is still challenging because classic transgenic mouse methods are not as applicable to non-coding regions [18,44-47]. Allele-specific expression (ASE) analysis offers a powerful approach to distinguishing pathogenic regulatory variants from benign polymorphisms. By phasing RNA-seq reads to specific haplotypes, it is possible to determine whether a variant allele is associated with the reduced or aberrant expression of a nearby gene. For example, a non-coding enhancer variant that results in the monoallelic downregulation of TBX5 in cardiomyocytes provides strong evidence of pathogenicity. In contrast, variants that show balanced biallelic expression are less likely to be clinically relevant. This allele-level resolution strengthens causal inference for variants identified in non-coding regions [44-47].

Integrating multi-omic datasets enhances the resolution of gene discovery by linking genetic variation to molecular function and phenotype. Chromatin conformation tools such as Hi-C or Capture-C can further link enhancer variants to specific promoters, while proteomic data provide insight into downstream pathway perturbations. Recent work has implicated non-coding de novo variants in the aetiology of congenital heart disease, though the precise effects of such regulatory mutations on transcriptional control and cardiac development remain largely undefined [48].

Epigenetic Modifications

Epigenetic mechanisms may also be involved in gene silencing [18,49-52]. Dobosz et al. (2019) showed that the hypermethylation of the *NRG1* gene correlated with the presence of heart defects in Down syndrome [53]. Previously, Guo et al. (2015) demonstrated that histone modifier genes altered conotruncal heart phenotypes in 22q11.2 deletion syndrome [54]. Mosaicism is a rare cause of CHD (<2%) as a result of gene mutations early in embryogenesis that can result in two or more cell populations, each with a distinct genotype [18].

Another potential reason as to why there has been little progress in identifying novel CHD genes may be flawed designs of the large-scale genetic studies that have overlooked the biology and phenotype of the various CHDs. Despite recruiting a large number of patients into syndromic and non-syndromic groups, CHDs have been grouped together without due consideration to their phenotype or developmental biology [55]. Clubbing together such heterogeneous categories of CHD lends itself to drawing erroneous conclusions, and such approaches are unlikely to contribute to our understanding of CHD.

*Improving the *‘*Hit Rate*’* for Finding CHD-Causing Genes*

To enhance the success rate of identifying CHD-causing genes, a refined and systematic approach is needed. Genetic studies must begin with rigorous phenotyping, grouping both syndromic and non-syndromic CHDs based on clearly defined, specific clinical features. Patient classification should be consistent, unbiased and informed by developmental biology [9]. Subtypes of CHD should be diagnosed according to established clinical criteria. Detailed multigenerational family histories, including instances of early or unexplained deaths, are critical.

Karyotyping and chromosomal microarray (CMA) remain valuable for detecting aneuploidies and copy number variants, though they cannot identify balanced rearrangements or small sequence changes. Karyotyping has low resolution, typically detecting only abnormalities larger than 5-10 Mb. CMA improves on resolution but does not detect balanced translocations or most single-nucleotide variants. Whole-exome sequencing (WES) offers cost-effective, high-resolution interrogation of coding regions but may miss non-coding and structural variants. WES is confined to coding exons and is susceptible to coverage gaps, particularly in guanine-and-cytosine-rich regions. Whole-genome sequencing (WGS) provides broader coverage, capturing regulatory variants and structural changes, though interpretation remains challenging. Even long-read sequencing, though powerful, remains limited by throughput and cost. Moreover, commonly used molecular techniques such as polymerase chain reaction (PCR) (including quantitative PCR {qPCR} and allele-specific PCR), high-resolution melting analysis and microarray-based genotyping can occasionally yield false-positive or false-negative results due to issues such as contamination, non-specific amplification, probe misbinding or low signal-to-noise ratios. By acknowledging these limitations, we emphasise that robust gene discovery relies on integrating multiple technologies, each addressing different variant classes. Collecting DNA samples from as many affected and unaffected relatives as possible further strengthens the genetic analysis.

Overall, while large-scale chromosomal abnormalities can be detected using karyotyping, array comparative genomic hybridisation (CGH) and fluorescence in situ hybridisation (FISH), smaller variants such as single-nucleotide changes or small insertions/deletions require more advanced techniques such as targeted gene panels, WES or WGS [2,18]. WGS performed with long-read technologies not only enables the detection of single-nucleotide polymorphisms (SNPs) and CNVs across extended DNA regions but also provides superior accuracy in characterising pseudogenes and repetitive regions when compared with short-read WGS (Table 1) [56].

**Table 1:** Current genetic techniques and their applications in the context of CHD CNVs, copy number variants; SNVs, single-nucleotide variants; SNPs, single-nucleotide polymorphisms; CHD, congenital heart disease

Technique	Application	Examples of CHD detected
Karyotype	Aneuploidy and translocations	CHDs associated with Trisomy 21, 18 and 13; monosomy X
Comparative genomic hybridisation (CGH)/single-nucleotide polymorphism (SNP) Array	Large unbalanced CNVs and genomic rearrangements	CHDs associated with DiGeorge syndrome
Fluorescent in situ hybridisation (FISH)	Deletion/duplication of specific DNA sites	CHDs associated with Trisomy 21, 18 and 13; DiGeorge syndrome
Multiplex ligation-dependent probe amplification	Microdeletion and CNVs	DiGeorge syndrome; 1p36 deletion syndrome
Chromosomal microarray analysis (CMA)	Chromosomal anomalies and CNVs	DiGeorge syndrome
Whole-exome sequencing	SNVs, microdeletions and protein-coding regions	Tetralogy of Fallot and patent ductus arteriosus
Gene panel testing	SNPs and CNVs	Monogenic CHDs and tetralogy of Fallot (CNVs are a part of some panels)
Whole-genome sequencing	SNVs, insertions, microdeletions and non-coding and protein-coding regions	Broadest genetic test available that can detect a number of CHDs
Fetal cell-free DNA testing	Non-cellular fetal DNA	CHDs associated with Trisomy 21, 18, and 13; monosomy X

Emerging and Discovery-Stage Technologies

While clinically approved genetic tests remain central to the evaluation of CHDs, a number of advanced technologies are currently being explored in research laboratories. These discovery-stage approaches are not yet part of routine clinical practice but provide valuable insights into the genetic and molecular basis of CHDs.

Human pluripotent stem cells (hPSCs) offer a particularly powerful research platform for modeling CHDs, especially non-syndromic forms driven by single-gene defects. By generating patient-specific induced pluripotent stem cells (iPSCs), researchers can recapitulate cardiogenesis in vitro, enabling the study of disease mechanisms at the cellular and molecular levels [57]. Long-read sequencing technologies represent another promising area of innovation. Compared with short-read sequencing, long-read approaches improve the resolution of pseudogenes, repetitive regions and complex structural variants that are often missed in standard analyses. Although currently limited by cost and accessibility, ongoing improvements in accuracy and scalability suggest that long-read sequencing may eventually complement or replace short-read whole-genome sequencing in the clinical setting [58]. Finally, CRISPR/Cas-based genome editing is increasingly used as a research tool to validate candidate genes and dissect pathogenic pathways in CHD [59,60].

When combined with hPSC-derived cardiomyocyte models, genome editing enables the direct assessment of variant pathogenicity and functional impact. These strategies highlight the growing translational bridge between discovery research and precision cardiovascular medicine [61-65].

## Conclusions

CHDs result from perturbations in the complex transcriptional and signalling networks that guide heart development. Key transcription factors and signalling molecules coordinate cellular behaviours essential for morphogenesis, and their disruption leads to a broad spectrum of cardiac anomalies. The underlying causes of CHD appear to be multifactorial, with a strong genetic component where many genes in cardiac development likely overlap with CHD.

Although WES and WGS have led to the discovery of several new genes, accurate phenotyping in CHD studies is needed to establish the pathogenicity of identified variants. Advances in single-cell genomics, stem cell biology and genome editing are offering unprecedented insight into the molecular mechanisms underlying CHD. The continued integration of these tools with clinical phenotyping will be essential for improving genetic diagnosis, guiding therapeutic interventions and ultimately reducing the burden of CHDs.

## Appendices

Table 2 shows congenital cardiac anomalies by developmental stage and genes.

**Table 2:** Congenital cardiac anomalies by developmental stage and genes TAPVC, total anomalous pulmonary venous connection; AV, atrioventricular; LV, left ventricle; LA, left atrium

Congenital cardiac anomaly	Cardiac developmental stage of arrest	Genes involved	References
Tetralogy of Fallot (TOF)	Outflow tract (OFT) septation and cardiac neural crest migration/alignment	*TBX1* (22q11.2), *NOTCH1*, *FLT4*/*VEGFR3* and *JAG1*	Matos-Nieves et al. (2019) [66]; Page et al. (2019) [67]; Huang et al. (2013) [68]; Althali and Hentges (2022) [69]
D-Transposition of the great arteries (D-TGA)	Left-right laterality and OFT alignment/rotation	*NODAL*, *ZIC3* and *CFC1* (plus ciliary genes in some cohorts)	Škorić-Milosavljević et al. (2022) [70]; Zubrzycki et al. (2024) [71]; Dardas et al. (2024) [72]
Persistent truncus arteriosus (PTA)	OFT septation failure (conotruncal cushions; neural crest)	*TBX1* (22q11.2) and *GATA6*	Yamagishi (2024) [73]; Yasuhara et al. (2024) [74]
Double-outlet right ventricle (DORV)	OFT alignment (conal/infundibular alignment; second heart field)	*ZFPM2*/*FOG2*, *GATA4* and *GATA6*	Bolunduț et al. (2023) [75]
Ventricular septal defect (membranous)	Endocardial cushion-to-ventricular septation (membranous septum)	*GATA4*, *NKX2-5* and *TBX5*	Cervantes-Salazar et al. (2024) [76]; Chaithra et al. (2022) [77]; Al Zubaidi et al. (2024) [78]
Atrial septal defect (secundum)	Atrial septation (septum primum/secundum formation)	*GATA4*, *NKX2-5*, *TBX5* and *MYH6*	Cervantes-Salazar et al. (2024) [76]; Chaithra et al. (2022) [77]; Al Zubaidi et al. (2024) [78]
Atrioventricular septal defect (AVSD)	Endocardial cushion (AV canal)/endocardial-to-mesenchymal transition	*CRELD1* (plus Down syndrome-related modifiers)	Asim et al. (2018) [79]; Ramachandran et al. (2015) [80]
Bicuspid aortic valve (BAV)	Semilunar valve (aortic) cusp formation and OFT cushion remodeling	*NOTCH1*, *SMAD6*, *ROBO4* and *GATA5/6*	Debiec et al. (2022) [81]; Mansoorshahi et al. (2024) [82]; Moosa and Garcia (2025) [83]
Coarctation of the aorta (CoA)	Aortic arch artery remodeling (pharyngeal arch derivatives)	*NOTCH1* (±*ELN* in syndromic forms)	Parker and Landstrom (2021) [84]; Salciccioli and Zachariah (2023) [85]; Kerstjens-Frederikse et al. (2016) [86]
Hypoplastic left heart syndrome (HLHS)	Second heart field proliferation and LV outflow/chamber formation	*NOTCH1* and *MYH6*	Theis et al. (2021) [87]; Tomita-Mitchell et al. (2016) [88]; Quintanilla Anfinson et al. (2024) [89]; Sergi (2025) [90]
Ebstein anomaly (tricuspid)	AV valve delamination/morphogenesis (failure of leaflet delamination)	*MYH7* and *NKX2-5*	Sedaghat-Hamedani et al. (2017) [91]; Ritter et al. (2022) [92]
Patent ductus arteriosus (PDA)	Ductus arteriosus smooth-muscle constriction and postnatal remodeling	*TFAP2B* (Char syndrome) and *PTGIS* (modifiers)	Fernandez Trahan et al. (2025) [93]; Gillam-Krakauer and Mahajan (2023) [94]; Lewis et al. (2018) [95]
Total anomalous pulmonary venous return (TAPVR/TAPVC)	Failure of common pulmonary vein incorporation into LA	*SEMA3D*, *ZDHHC8* and *HMGA2* (heterogeneous)	Shi et al. (2018) [96]; Poelmann et al. (2024) [97]
Pulmonary valve stenosis	Semilunar valve (pulmonary) cusp formation	*JAG1*/*NOTCH2* (Alagille) and *GATA4* (subset)	Marchini et al. (2023) [98]

Table 3 shows genes and associated congenital heart diseases.

**Table 3:** Genes and associated congenital heart diseases VSD, ventricular septal defect; HLHS, hypoplastic left heart syndrome; LVOT, left ventricular outflow tract; ASD, atrial septal defect; AVSD, atrioventricular septal defect; PTA, persistent truncus arteriosus; DORV, double-outlet right ventricle; BAV, bicuspid aortic valve; AV, atrioventricular; LV, left ventricle; D-TGA, D-transposition of the great arteries; TAPVR/TAPVC, total anomalous pulmonary venous return/connection; CoA, coarctation of the aorta; HCM, hypertrophic cardiomyopathy

Name of gene	Examples of CHDs/cardiac malformations	References
TBX1	Tetralogy of Fallot (TOF), persistent truncus arteriosus, interrupted aortic arch, VSD and conotruncal anomalies	Yamagishi (2024) [73]; Althali and Hentges (2022) [69]; Gould et al. (2019) [99]
NOTCH1	Bicuspid aortic valve, coarctation, HLHS, LVOT obstructive lesions, tetralogy of Fallot and aortic valve disease	Debiec et al. (2022) [81]; Parker and Landstrom (2021) [84]
GATA4	ASD (secundum), VSD, AVSD, TOF and pulmonary stenosis	Chaithra et al. (2022) [77]; Cervantes-Salazar et al. (2024) [76]
GATA6	PTA, DORV, VSD, OFT malformations and BAV	Yasuhara et al. (2023) [74]
NKX2-5	ASD, VSD, Ebstein anomaly and AV block association	Al Zubaidi et al. (2024) [78]
TBX5	ASD, VSD, Holt-Oram syndrome spectrum and conduction defects	Cervantes-Salazar et al. (2024) [76]; Chaithra et al. (2022) [77]
MYH6	ASD, VSD and HLHS	Theis et al. (2021) [87]; Tomita-Mitchell et al. (2016) [88]; Chaithra et al. (2022) [77]
MYH7	Ebstein anomaly, LV noncompaction and HCM (overlap)	Ritter et al. (2022) [92]
*JAG1*/*NOTCH2*	Pulmonary stenosis, TOF, peripheral pulmonary artery stenosis and Alagille syndrome spectrum	Gilbert et al. (2024) [98]
SMAD6	BAV, LVOT obstruction and aortic valve disease	Debiec et al. (2022) [81]
ROBO4	BAV and aortic valve disease	Bruenger et al. (2025) [100]
*NODAL*/*CFC1*/*ZIC3*	Heterotaxy, D-TGA and laterality defects	Dardas et al. (2024) [72]; Zubrzycki et al. (2024) [71]
CRELD1	AVSD (especially Down syndrome-related)	Asim et al. (2018) [79]; Ramachandran et al. (2015) [80]
TFAP2B	Patent ductus arteriosus (Char syndrome)	Fernandez Trahan et al. (2025) [93]; Gillam-Krakauer and Mahajan (2023) [94]
*SEMA3D*, *ZDHHC8* and *HMGA2*	TAPVR/TAPVC	Shi et al. (2018) [96]; Poelmann et al. (2024) [98]
ELN	Supravalvular aortic stenosis and CoA (Williams syndrome)	Parker and Landstrom (2021) [84]; Salciccioli and Zachariah (2023) [85]
